# Efficacy and safety of endoscopic sphincterotomy with balloon dilation versus endoscopic sphincterotomy alone for extraction of common bile duct stones with a maximum diameter of 10 to15 millimeters

**DOI:** 10.22088/cjim.14.2.226

**Published:** 2023

**Authors:** Mehdi Yalmeh, Abdolreza Emami, Javad Shokri Shirvani, Seyyed Hassan Abedi Valukalaei, Mohamadtaghi Hamidian, Hemmat Gholinia Ahangar

**Affiliations:** 1 Student Research Committee, School of Medicine, Babol University of Medical Sciences, Babol, Iran; 2 Department of Internal Medicine, School of Medicine, Babol University of Medical Sciences, Babol, Iran; 3 Health Research Institute, Babol University of Medical Sciences, Babol, Iran

**Keywords:** Endoscopic retrograde cholangiopancreatography, Common bile duct stone, Efficacy, Safety, Endoscopic Sphincterotomy, Endoscopic sphincterotomy with balloon dilation

## Abstract

**Background:**

Various factors, most notably the stone's features, determine the selection of an appropriate method to extract common bile duct (CBD) stones during endoscopic retrograde cholangiopancreatography. In this study, the efficacy and safety of endoscopic sphincterotomy with balloon dilation (ESBD) versus endoscopic sphincterotomy (EST) for CBD stone extraction with a diameter of 10 to 15 millimeters were compared.

**Methods:**

This retrospective cross-sectional study included 154 patients referred to the Rouhani Hospital in Babol, Iran, with CBD stones. Consensus sampling was used. Each individual's demographic information and findings from the procedure were entered into the SPSS software (v. 26). A level of less than 0.05 was considered statistically significant.

**Results:**

A total of 154 patients were included in the study, of which 81 (52.6%) were in the EST, and 73 (47.4%) were in the ESBD group. Complete stones removal rate was higher in the ESBD versus the EST group (79.5% versus 46.9%, P<0.001). No significant differences were observed between the two methods' overall side effects rate (P = 0.469).

**Conclusion:**

For the complete extraction of CBD stones larger than 10 millimeters, the ESBD method outperforms the EST method.

Due to the high prevalence of common bile duct (CBD) stones, surgical complications, and advancements in endoscopic techniques, endoscopic retrograde cholangiopancreatography (ERCP) is widely used as the primary method for diagnosing and treating CBD stones (1). In fact, the European Society of Gastrointestinal Endoscopy (ESGE) and the American Society for Gastrointestinal Endoscopy (ASGE) both strongly recommend that patients with CBD stones undergo ERCP (2, 3). Since 1975, several endoscopic techniques for extracting CBD stones have been published in the literature, including endoscopic sphincterotomy (EST), endoscopic papillary balloon dilation (EPBD), endoscopic sphincterotomy with balloon dilation (ESBD), endoscopic sphincterotomy plus large balloon dilatation (ES-LBD), and mechanical lithotripsy. Naturally, each has its own set of indications, benefits, and complications (4).

The method of stone extraction is primarily determined by the stone's characteristics, the patient's comorbidities, the availability of equipment, and the physician's preferences. However, stone characteristics are more significant than others (2). While there is consensus that multiple interventions are required to extract large CBD stones (2, 5), the ESGE and ASGE guidelines disagree on the definition of large stones. Indeed, the ESGE considers stones with a diameter of ≥ 15 millimeters (2), whereas the ASGE defines large CBD stones with a diameter of ≥ 10 millimeters (3).

Previous research established that the ESBD method is superior to the EST method in patients with large CBD stones (1, 2, 4, 6). For example, Dong et al. (1) demonstrated that the ESBD group had a statistically higher removal rate of ≥ 10-millimeter CBD stones than the EST group during the first ERCP session (OR 2.07; 95% CI; 1.37 to 3.12), but a significantly higher removal rate of ≥ 15-millimeter CBD stones was not observed. Additionally, the ESBD method had a lower rate of complications than the EST method (OR 0.63; 95% CI; 0.47 to 0.85).

Xu et al. (7) classified ≥ 10-millimeter CBD stones as difficult stones and demonstrated that the EPLBD+mEST method was superior to the EST method for complete stone extraction (94.5% vs. 84.2%, P=0.04). However, due to the discrepancy in the definition of large stones, there is debate over the cut-off point for defining large CBD stones and selecting the most effective treatment approaches. Thus, the purpose of this retrospective cross-sectional study was to compare the efficacy and safety of the ESBD versus the EST method for extracting CBD stones with a diameter of 10 to 15 millimeters in order to establish a more precise definition for large CBD stones and, consequently, the most appropriate treatment option based on their size.

## Methods

This was a retrospective cross-sectional study of patients with a known case of CBD stones referred to the Rouhani Hospital in Babol, Iran, for further evaluation and treatment from March to July 2021. The study protocol was reviewed and approved by both the institutional review board and the ethics committee of Babol University of Medical Sciences (IR.MUBABOL.REC.1399.507). Each patient's prepared form contained all information necessary, including demographic data, previous imaging studies, and findings during the ERCP session. 

Inclusion criteria were as follows: 1. Age ≥ 18 years old; 2. Presence of CBD stones with a diameter of 10 to 15 millimeters in imaging studies before the ERCP session; 3. Absence of intra-hepatic biliary stones in imaging studies before the ERCP session; 4. Absence of coagulation disorder in patient's laboratory evaluations before the ERCP session (defined as platelet count < 50000 per microliter of blood); 5. No history of anti-coagulation drug use within a week before the ERCP session; 6. No history of previous ERCP sessions. 

The exclusion criteria were as follows: 1. Presence of stenosis in the distal segment of the CBD or susceptibility to a malignant lesion in the CBD as a result of ERCP findings; 2. Any life-threatening event that necessitated the ERCP session's early termination. The determining variable was whether the EST or ESBD was used to extract stones during the ERCP session. The primary outcome was complete CBD stone extraction, confirmed via direct fluoroscopy by an experienced endoscopist. Secondary outcomes included the following: 1. Any complications occurred during the ERCP session or for up to 24 hours afterward; 2. The total duration of the ERCP session; 3. The need for mechanical lithotripsy. 

We used the study conducted by Xu et al. (6) to determine the minimum sample size required, with the primary outcome being complete CBD stone extraction. The calculations demonstrated that with an 80% power and a 5% first type error, we required at least 75 patients in each group to detect a 10.3% difference in the rate of CBD stone extraction between the two methods. All completed forms were entered into the SPSS software (version 26) for further statistical analysis. For qualitative variables, frequency and percentage were used to describe data tendency; for quantitative variables, mean and standard deviation were used. The Chi-square and independent student T-tests were used to determine the possibility of a relationship between variables. A value of less than 0.05 was considered statistically significant in all statistical analyses.

## Results

In the final analysis, 154 patients were recruited, with 81 (52.6%) using the EST method and 73 (47.4%) using the ESBD method for CBD stone extraction. As shown in Table 1, there were no significant differences between the study groups in patient characteristics or imaging findings prior to the ERCP session.

Complete CBD stone extraction was determined for both groups, and as shown in Table 2, the ESBD method extracted complete CBD stones significantly more efficiently than the EST method (P<0.001). Indeed, the odds ratio for complete CBD stone extraction using the ESBD and EST methods was 2.33 and 0.53, respectively. In other words, the ESBD method was more efficient than the EST method for complete CBD stone extraction. No mechanical lithotripsy was performed on our patients to facilitate stone extraction regardless of the method selected. As shown in Table 3, the ESBD method had a higher overall side effect rate than the EST method (16.4 % vs.12.3%), but this difference was not statistically significant (P=0.469). Finally, we observed that the ESBD method required additional time to complete the ERCP session than the EST method (11.83 vs. 9.50 minutes, respectively, P<0.001).

**Table 1:** Comparison of patients’ characteristics and findings of imaging studies before the ERCP session in the ESBD and EST groups

Variables		ESBD method (n=73)	EST method (n=81)	P-value
Age in years (mean ± SD)		59.58 ± 17.23	58.12 ± 19.65	0.628
Gender (frequency, percentage)	**Female**	37 (50.7)	37 (45.7)	0.535
	**Male**	36 (49.3)	44 (54.3)	
Presence of GB stone (frequency, percentage)		26 (35.6)	36 (44.4)	0.265
Max diameter of CBD in millimeters (mean ± SD)		11.04 (2.53)	12.00 (7.22)	0.294
Max diameter of CBD stones in millimeters (mean ± SD)		11.26 (2.36)	11.02 (1.49)	0.466
Total number of CBD stones in each patient (frequency, percentage)	**≤ 3**	68 (93.2)	74 (91.4)	0.228
	**3 to 5**	5 (6.8)	4 (4.9)	
	**≥ 5**	0 (0.0)	3 (3.7)	

SD=standard deviation, GB=gall bladder, max=maximum, SD=standard deviation

**Table 2:** Comparison of complete CBD stone extraction in the ESBD the EST groups

	Complete CBD stone extraction (frequency, percentage)		P-value	Odd Ratio	CI 95%	
	Yes	No			Lower	Upper
ESBD method (n=73)	58 (79.5)	15 (20.5)	<0.001*	2.33	1.46	3.71
EST method (n=81)	38 (46.9)	43 (53.1)		0.53	0.39	0.71

CI=confidence interval, *: Chi-Square Test

**Table 3:** Comparison of ERCP-related complications in the ESBD and EST groups

Side effect (frequency, percentage)	ESBD method (n=73)	EST method (n=81)	P-value	OR (ESBD/ EST)	CI 95%	
					Lower	Upper
Cholangitis	0 (0)	1 (1.2)	0.341	--	--	--
Perforation	1 (1.4)	0 (0)	0.291	--	--	--
Bleeding	1 (1.4)	2 (2.5)	0.622	0.54	0.04	6.18
post-ERCP pancreatitis	10 (13.7)	7 (8.6)	0.317	1.67	0.60	4.66
Total	12 (16.4)	10 (12.3)	0.469	1.39	0.56	3.45

OR=odds ratio

## Discussion

Most Previous studies (2, 8, 9) demonstrate that the EST method is more effective than the ESBD method for extracting ≥ 10-millimeter CBD stones without significantly increasing the risk of ERCP-related complications. Moreover, multiple studies demonstrate that the ESBD method is more efficient and safer than the EST method for extracting ≥ 15-millimeter CBD stones, whether during the initial ERCP session (1, 4, 7, 10-15) or multiple ERCP sessions (16, 17). The ESGE recommends using ESBD instead of EST for extracting ≥ 15-millimeter CBD stones due to its increased effectiveness and safety (2). In comparison, the ASGE notes that the ES-LBD is more effective than EST at extracting ≥ 10-millimeter CBD stones without causing significant ERCP-related side effects (3). Our findings support the ASGE recommendation to use the ESBD method as a first-line method for extracting ≥ 10-millimeter CBD stones (3).

Previous research indicates that the ESBD requires significantly less mechanical lithotripsy to achieve complete CBD clearance when dealing with ≥ 15-millimeter CBD stones (10, 16, 18), whereas neither the EST nor the ESBD group received mechanical lithotripsy in our study. One possible explanation for this controversy is that CBD stones ≥ 15 millimeters typically require additional intervention such as ES-LBD or mechanical lithotripsy to be removed (2, 5), whereas our study excluded patients with CBD stones ≥ 15 millimeters. The absence of a single method for determining the size of stones, a retrograde study, a short follow-up period, and a lack of information about patients who had unsuccessful ERCP were several of the study's limitations. In summary, our findings confirm the ASGE recommendation regarding the high efficacy of the ESBD method for the extraction of ≥ 10-millimeter CBD stones, and we suggest using a 10-millimeter cut-off for difficult CBD stones instead of the 15-millimeter cut-off in the ESGE recommendation.
